# Innovative Individuals Are Not Always the Best Demonstrators: Feeding Innovation and Social Transmission in *Serinus canaria*

**DOI:** 10.1371/journal.pone.0008841

**Published:** 2010-01-22

**Authors:** Nicole Cadieu, Stéphane Fruchard, Jean-Claude Cadieu

**Affiliations:** 1 UPS, Centre de Recherches sur la Cognition Animale, Université de Toulouse, Toulouse, France; 2 CNRS, Centre de Recherches sur la Cognition Animale, Toulouse, France; Queen Mary University of London, United Kingdom

## Abstract

**Background:**

Feeding innovation occurs when individuals choose a novel, unknown type of food and/or acquire new feeding skills. Here we studied feeding innovation and social transmission of the new feeding habit in canaries. Adult canaries eat a wide variety of seeds but avoid larger ones such as those of sunflowers. We determined whether adults of both sexes are equally prone to innovate when confronted with sunflower seeds and whether free-interactions facilitate transmission of the new feeding habit in a sex-dependent manner.

**Methodology/Principal Findings:**

First we determined which sex was more innovative, i.e., was more successful at husking and eating the novel seeds. Males were clearly more innovative than females. Due to this, experienced males served as model for either male or female observers in three different conditions (free interaction with a demonstrator, visual interaction with a demonstrator placed behind a transparent wall and access to seeds in the presence of a non-demonstrating bird). During free interactions, the new feeding habit was only transmitted to females. In contrast, transmission of seed handling to male observers only occurred if demonstrator and observer were separated by the transparent wall. Indeed, aggressive behaviors between males prevented social transmission during free interactions. Finally, we studied the influence of the less innovative females in feeding-habit transmission. First, we obtained female demonstrators by making them freely interact with male demonstrators. Once they acquired innovative responses to sunflower seeds we studied feeding-habit transmission towards male and female observers. Observers of both sexes learned during free interactions with female demonstrators. No aggressive behavior occurred. Males were also able to learn after visual interactions with the female demonstrator.

**Conclusions/Significance:**

Our results show that the most innovative individuals (males) are not always the best demonstrators, and that social relationship and sex are crucial factors for the spread of a new feeding habit among canaries. These factors determine the kind of interaction between individuals and the time spent together, thus affecting the transmission of novel habits within the population.

## Introduction

Innovation in animal behavior is defined as the process that results in new or modified learned behavior and that introduces novel behavioral variants into a population's repertoire [1]. Focusing on individual behavior, Ramsey et al. [2] stated that “innovation is the process that generates in an individual a novel learned behavior that is not simply a consequence of social learning or environmental induction”. Taken together, these definitions underline the role of innovation in the way that animals interact with their environment. Innovative behavior may indeed lead to new morphological, behavioral and physiological adaptations in animals [3].

Feeding innovation occurs when individuals choose a novel, unknown type of food and/or acquire new feeding skills [4]–[7]. Factors like personality traits [8], life period and age of the subjects [9]–[10] or food deprivation [7] may facilitate innovative feeding behavior in animals. In this context, determining which individuals are prone to innovate feeding habits in a population is particularly interesting.

Innovation and social transmission are not synonymous. While some individuals may innovate, others may facilitate spreading of new habits. In Japanese macaques, for instance, youngsters are more innovative when it comes to introducing innovative sweet potato washing. This new habit is then thought to have been transmitted to dominant individuals who spread it through the whole population [11]. Novel habit spreading may be oriented by an active choice of the model by observers and by interactions between tolerant, experienced subjects and naïve individuals [7;12–15]. In birds, various cases of innovation followed by social transmission have been observed in the field [7;16–17]. Analyses of the conditions required for a new habit to spread revealed contradictory results. In some cases, simultaneous access of demonstrator and observer to food prevents social learning [18]–[19] especially when the food produced is divisible and when scrounging is more profitable to the observer than learning. Social learning about food habits may also be prevented if the observers' attention is distracted by the reward made available by the model. In contrast, opposite effects, i.e. facilitation of social learning, may occur if one considers that accessing food will reinforce the acquisition of new feeding habits [20]–[22]. Moreover, if the reward is spatially related to the place where the feeding habit is learned, birds will also exhibit higher levels of social learning, especially if a high degree of tolerance exists between demonstrators and observers [23].

In canaries, controlled laboratory experiments showed that feeding habit transmission occurs from experienced adults to familiar juveniles [15], [23]–[24]. Adult males, which look after their progeny after fledging, serve as a food-choice and food-handling model for juveniles. The question of whether demonstrating canaries are also those innovating when it comes to choosing and handling a new food has not been studied so far. Here we studied innovation in adult canaries in the presence of an unknown food. Adult domestic canaries eat a wide variety of seeds but avoid larger ones, such as those of sunflowers (*Helianthus annuus*) [25]. Wild canaries are resident birds which live exclusively on Atlantic islands (Azores, Canary Islands, Madeira) where sunflowers, originated in Mesoamerica, were not originally available. Thus, sunflower seeds do not belong to the original wild diet of these birds, which consume smaller seeds of annual grass species (*Avena sterilis, Lolium lowei*), Ice plant (*Mesembryanthemum crytallinum*), or annual succulents (*Chenopodium murale* and *Sonchus oleraceus*) [26]. Yet, wild canaries forage on food patches and this situation may favor social learning. Thus, if birds were prone to innovate their feeding habits, and this innovation would spread in the population, wild birds could eventually also forage on novel seeds such as those of sunflowers.

Feeding on a novel seed requires its recognition as food and the acquisition of an efficient handling skill. We determined whether adult individuals of both sexes are equally prone to innovate when confronted with sunflower seeds and whether free-interactions facilitate transmission of the new feeding habit in a sex-dependent manner. Our results thus aim at answering the question of whether innovative individuals are also those facilitating social transmission, a fundamental question in studies on social behavior in animals.

## Materials and Methods

All the experimental procedures comply with French laws governing experiments on animals. Experiments on canaries were carried out in our laboratory under license from the French Ministry of Agriculture and Forestry. The birds did not lose weight during the experiment and were housed in individual cages to avoid aggression.

In a *first experiment*, we studied innovative feeding behavior of adult male and female canaries in the presence of unknown sunflower seeds. In a *second experiment*, we determined whether male and female canaries transmit the new feeding habit to other birds, either males or females.

We used adult canaries between 10 and 26 months old. The subjects were domestic birds hatched in our laboratory. Birds were fed with a commercial mixture of small canary seeds, mash and greenery and had no experience of sunflower seeds. Before the experiments, birds were housed individually in a breeding room, in cages of 60×30 cm and 35 cm high. They were kept at 25±1°C under a 15∶9 h light: dark cycle (corresponding to breeding period). Irrespective of sex and of experiment, birds did neither differ in weight, nor in bill length (which provides a reliable estimation of a bird's strength), nor in age (see Table 1).

**Table 1:** Age, weight and bill size of canaries (males and females) in Experiments 1 and 2.

	Experiment 1		Experiment 2Male Demonstrators		Experiment 2Female Demonstrators		
Subjects	Males(n = 20)	Females(n = 20)	Males(n = 20)	Females(n = 20)	Males(n = 20)	Females(n = 20)	p-Value
**Age**	17,2±5	14,6±4	16,8±3	18,1±5	16,3±4	16,0±5	Sex: *>0.05*Experiment: *>0.05*Interaction: *>0.05*
**Weight**	27,3±3.1	26,8±3	26,5±2	25,6±2	26,7±3	27,0±3	Sex: *>0.05*Experiment: *>0.05S*Interaction: *>0.05*
**Bill size**	10,6±0.6	10,4±0.7	10,5±0.7	10,0±0.8	10,3±0.8	10,1±0.5	Sex: *>0.05*Experiment: *>0.05*Interaction: *>0.05*

Data are means ± SD.

### 1) Experiment 1: Which Sex Is the Most Innovative One?

Birds of each sex were transferred to the experimental room and visually isolated in individual cages 60×30×35 cm where the unknown sunflower seeds were presented in a 20 cm^3^ feeder. Intact, large sunflower seeds (10 mm length in average) were used so that birds had to husk them in order to ingest them. In order to avoid undesired weight-losses due to confrontation with a novel food, birds were fed with mash and water during a period of 14 days during which they learned to manipulate and eat sunflower seeds (experimental group: familiar with sunflower seeds, FS). The feeder containing sunflower seeds was daily changed. Another group was treated similarly but did not receive any seed during the same period (control group: NFS). After the 14–day period, all birds were fed with mash for 48 hours. Afterwards, their behavior towards sunflower seeds and mash was individually recorded during 90 min by means of digital video cameras. We recorded the number of birds of each sex which picked up and ate sunflower seeds after husking them, and the number of seeds picked up and eaten by each bird. This *first experiment* was conducted on 40 naïve birds (20 males and 20 females) divided equally between the two conditions, FS and NFS.

### 2) Experiment 2: Which Sex Is the Best Demonstrator?

#### Males as demonstrators

In this experiment, male canaries were used as demonstrators. We tested whether birds of the most innovative sex acted as efficient models for feeding habit transmission. These birds were deprived from sunflower seeds during 7 days after their 14–day familiarization period and were afterwards presented with seeds during 90 min. Birds managing to husk at least 7 seeds during this period were kept as demonstrators and fed only with mash until being exposed to a naïve bird (‘observer’) 24 hours later. Male and female observers had then the possibility to learn how to use sunflower seeds as food in three different conditions, each lasting 90 min.

Free interactions between demonstrator and observer during simultaneous access to seeds (*FI, free interactions*).
Visual interactions between a demonstrator eating sunflower seeds behind a transparent wall so that the observer could see but not access the seeds (*SD, simple demonstration*).
Access to seeds in the presence of a non-demonstrating bird located behind a transparent wall (*MP, mere presence*).

Comparing results from the MP condition with those obtained in either the FI or the SD condition allows uncovering the effects of demonstrating behavior of a model. Comparing results from the FI and the SD conditions reveals to what extent it is necessary for an observer to access the seeds with the model and the importance of free interactions.

In all cases, male and female observers were individually transferred to the recording cage 24 h before the introduction of the demonstrator in order to familiarize them with the new surroundings. The demonstrator was brought into the cage 10 min before the start of the experiment. A different demonstrator was used for each observer**.** The cage was fitted with 4 parallel perches 12 cm apart. Drinking water and mash were placed at each end. In the free interaction condition (FI), both the demonstrator and the observer could freely move and interact within the cage; a feeder filled with sunflower seeds was located between the two central perches; in the other two experimental conditions, a transparent Plexiglas wall placed between the 2 central perches bisected the cage. In the simple-demonstration condition (SD), the demonstrator accessed the feeder with sunflower seeds located next to the transparent divider; the observer was placed in the next compartment and could only observe how the demonstrator's behavior towards the seeds. In the mere-presence condition (MP), the two birds were separated by a transparent wall and only the naïve bird had access to sunflower seeds.

In the FI condition, we recorded the number of agonistic interactions, the time spent together at the feeder, and the number of seeds picked up and eaten after husking both by the demonstrator and by the observer. In the SD and MP conditions, we recorded the number of seeds picked up and eaten after husking by the bird which had access to the seeds. The number of observers that manipulated and consumed seeds was also recorded.

To check whether observers acquired the new feeding habit in the three experimental conditions, they were afterwards transferred to an unfamiliar, visually isolated cage 30×30 cm and 35 cm high. The cage contained mash and drinking water but no sunflower seeds. After 1 h, a 20 cm^3^ feeder filled with sunflower seeds was presented for 24 h. For each bird, we then recorded the number of seeds picked up and eaten; we also quantified the number of birds which picked up and ate seeds after each experimental treatment (FI, SD, MP). Sixty observers (30 males and 30 females) divided equally between the three conditions were used in this experiment.

#### Females as demonstrators

In this experiment, female canaries were used as demonstrators. We tested whether birds of the less innovative sex contribute to the spread of the new feeding habit in the population. To this end, females were first deprived of seeds and fed with mash during 7 days and then presented with sunflower seeds during 90 min in the presence of an innovative male which had already learned to manipulate and husk these seeds. A different demonstrator was used for each observer. The two birds could freely interact and accessed simultaneously the feeder containing sunflower seeds. Three of such sessions (once a day) were performed consecutively. Females were then kept for 24 hours in the presence of mash and sunflower seeds. After 7 days of seed deprivation, they were tested during 90 min in the presence of sunflower seeds. Females that managed to husk at least 7 seeds during this period were selected as demonstrators for observers, males or females.

The female demonstrator and the observer were then studied using the same procedure and experimental conditions (FI, SD and MP) of the experiment in which males acted as demonstrators. Variables recorded were also the same. Sixty observers (30 males and 30 females), divided equally between the three conditions, were used in this experiment.

### Statistics

To compare performance between sexes, a 2×2 Chi-square with Yates correction was used. To analyze variations in a specific variable (e.g. ‘number of seed husked’ or ‘eaten’) as depending on sex and experimental condition, ANOVA was used. To this end, data were transformed for normality when necessary. Scheffe's test for multiple contrasts was used for post-hoc analyses. Each contrast was separately assessed with its associated F-test [27]. When only two groups had to be compared, Student's t test was used [28]. In this case, the significance level was 0.05; otherwise it was 0.05 divided by the number of groups involved in the comparisons.

## Results

### 1) Experiment 1: Which Sex Is the Most Innovative One?

Adults of both sexes exhibiting the same age, weight, and bill size, were presented with the unknown sunflower seeds during a period of 14 days. During this period, birds of the FS group were allowed to manipulate and to eat seeds, while in the NFS group (control group) no seeds were available to the birds. Generally, both sexes in the FS group manipulated seeds (2×2 Chi-square after Yates correction P>0.05) but only males (10 out of 10) managed to eat them. When subsequently tested during 90 min, all ten males in group FS picked up the sunflower seeds, and nine of them husked and ate the seeds. In average, males manipulated 91±18 seeds (mean ± SE) from which they ate 12±3. Only one female out of ten manipulated and husked seeds. This female only picked up 12 seeds and ate 2 of them. Therefore, males and females significantly differed in their ability to husk seeds (2×2 Chi-square after Yates correction = 9.8, *P*<0.002). In group NFS, which was unfamiliar with sunflower, no bird picked up the unknown seeds. This shows that the innovative behavior of handling sunflower seeds resulted from individual experience and was circumscribed mostly to males.

### 2) Experiment 2: Which Sex Is the Best Demonstrator?

#### Males as demonstrators

**Figure 1:**
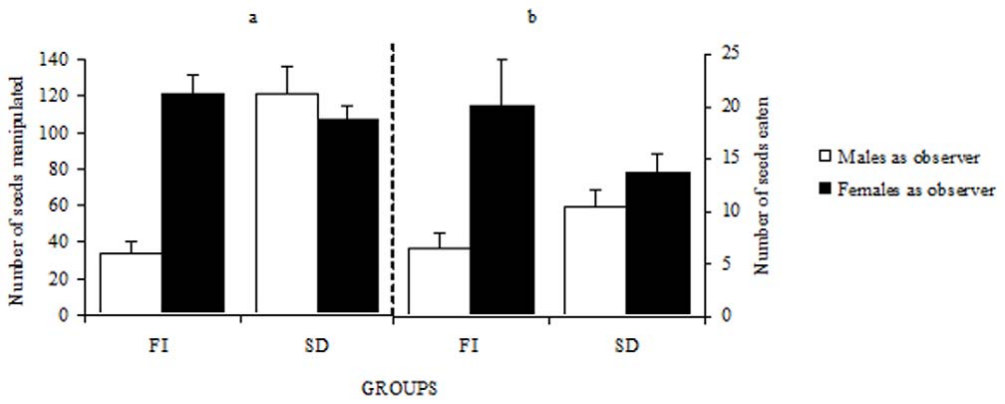
Males demonstrated more in the presence of female observers. Average (±SE) number of seeds picked up (a) and consumed after husking (b) by male demonstrator in the presence of an observer either male or female, when pairs of birds interacted freely (FI) and without access to seeds by the observer (SD).

We first focused on the demonstrating behavior of males towards observers in order to assess their contribution to the spread of the new feeding habit. Three experimental conditions were considered: FI, Free interaction between the demonstrator male and the observer; SD, male demonstrator and observer separated by a transparent wall (visual interactions) and MP, mere presence of a non-demonstrator male behind a transparent wall. The number of seeds manipulated by the male demonstrator (Fig. 1a) depended both on the sex of the observer (F1, 36 = 18.26, P<0.0001) and on the experimental conditions (F1,36 = 15.28, P<0.0004). The interaction between these factors was also significant (F1, 36 = 25.36, P<0.0001). In the FI condition, the male demonstrator manipulated fewer seeds in the presence of a male observer than in the presence of a female observer (Scheffé test: P<0.001), while in the SD condition no differences were detected (Scheffé test: P>0.05). The number of seeds eaten by the demonstrator (Fig. 1b) was only affected by the sex of the observer (F1, 36 = 11.08, P<0.002), but not by the experimental conditions (F1, 36 = 2.53, P>0.05.); i.e. male demonstrators husked and consumed more seeds in front of a female observer, irrespective of the experimental situation.

**Figure 2:**
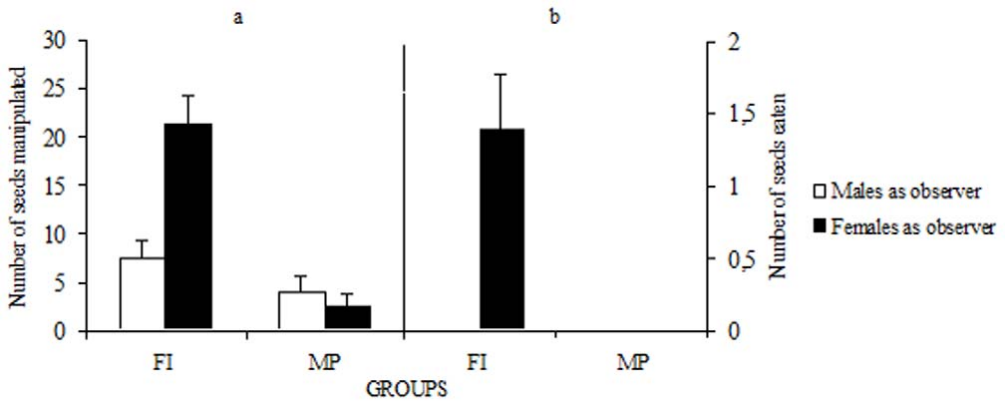
Free interactions with males favored handling only in females observers. Average (±SE) number of seeds picked up (a) and consumed after husking (b) by observers either male or female interacting freely with a male demonstrator (FI) and when a male was merely present (MP).

In the FI and the MP conditions, birds exposed either to a demonstrator or to a non demonstrating male were in direct contact with the seeds. Depending on the experimental conditions they behaved differently towards seeds (Fig. 2a; FI vs. MP, *F*_1, 36_ = 28.24, *P*<0.0001). Indeed, they manipulated more seeds in the FI condition than in the MP condition, i.e. when they could freely interact with the male demonstrator. A two-way ANOVA showed a significant interaction between sex and experimental condition (*F*_1, 36_ = 10.84, *P*<0.002). This was due to female observers picking up more sunflower seeds than male observers in the FI condition (*P*<0.01) while no such effect was found in the MP condition (*P*>0.05).

Manipulation translated into seed husking and consumption in 8 out of the 10 female observers which picked up seeds in the FI condition. In contrast, 9 out of 10 male observers did not husk the novel seeds in the presence of the male demonstrator, while 8 out of 10 picked up seeds (significant difference; 2×2 Chi-square after Yates correction = 7.2, *P*<0.01). In the presence of a non-demonstrating male (situation MP), 7 males and 5 females manipulated seeds, while only a single male consumed sunflower seeds (2×2 Chi-square after Yates correction P>0.05). Thus, an asymmetry in the number of males and females that manipulated and ate seeds existed only in the FI condition. This asymmetry was due to aggressive behavior (attacks) occurring in male-male interactions (attacks were observed in 8 out of 10 pairs; 8.3±1.81 attacks, mostly from the male demonstrator towards the male observer, occurred in 7 out of 8 pairs) but rarely in male-female interactions (2 out of 10 pairs; 0.3±0.21 attacks) where in one case, aggression came from the male demonstrator while in the other case it came from the female observer. This inter-sex difference affected the social coordination exhibited at the feeder. During male-male interactions, no coordination was observed between both partners. The two birds never visited the feeder simultaneously. In contrast, during male-female interactions, birds spent 34.4±5.3% of their total time at the feeder together. Tolerance at the feeder allowed female observers (9 out of 10) to eat small pieces of kernel dropped by the demonstrator or seeds partially opened by it. Male observers did not exhibit this behavior.

**Figure 3:**
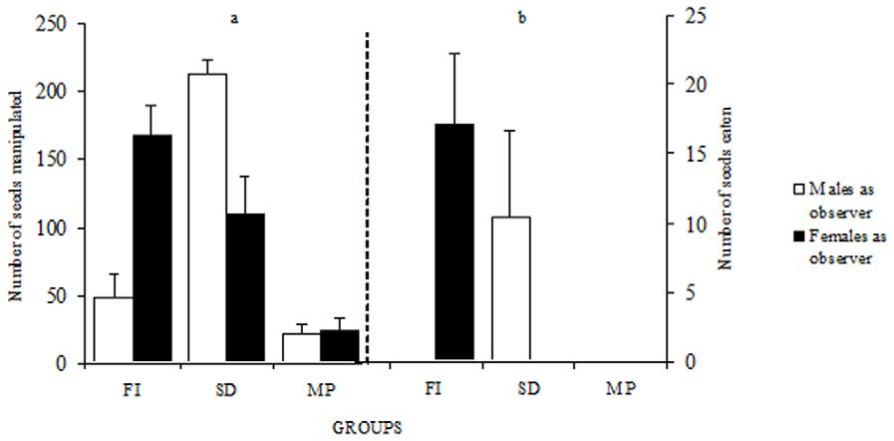
Complete social transmission from males occurred through different pathways in males and in females. Average (±SE) number of seeds picked up (a) and consumed after husking (b) by isolated male or female birds following the various interactions with a male demonstrator, when birds had previously interacted freely with the demonstrator (FI), after visual interactions (SD) and after access to seeds in the mere presence of a bird (MP). Free interactions were necessary for females, while visual interactions were sufficient for males. Aggressive behavior prevented social transmission between males during free interactions.

When males and female observers were subsequently isolated after the three experimental conditions (FI, SD and MP), the majority manipulated seeds. Only one male in the FI condition and one female in the MP condition did not pick up any seed. The number of manipulated seeds varied depending on the type of interactions to which birds were previously exposed (Fig. 3a; *F*_2, 54_ = 36.45, *P*<0.0001). Birds which had observed a demonstrator in action (FI and SD) manipulated more seeds than birds which had access to seeds in the presence of a non demonstrating bird (MP) (Scheffé test: *P*<0.005 for FI vs. MP and *P*<0.0005 for SD vs. MP). A significant interaction between sex and experimental condition was found (*F*_2, 54_ = 18.59, *P*<0.0001). Males manipulated significantly more seeds than females following the SD condition (Scheffé test: *P*<0.02), while the opposite trend was observed following the FI condition (Scheffé test: *P*<0.0001). This asymmetry determined that seeds were husked and eaten mostly by males following the SD condition and by females following the FI condition (Fig. 3b). After the MP condition, no seed consumption was observed in either sex. Following the FI condition, a single male ate seeds, while 10 out of 10 females consumed them. In contrast, following the SD condition, all males husked and ate seeds while no female showed these behaviors.

#### Females as demonstrators

We studied whether females facilitate social transmission of a new feeding habit despite their reduced tendency to innovate. In order to turn then in demonstrators, we exploited their capacity to learn from a male demonstrator. Once females acquired the new feeding habit, we studied whether they contribute to its spread the in the population by exposing them to observers, either males or females in the three conditions, FI, SD and MP.

**Figure 4:**
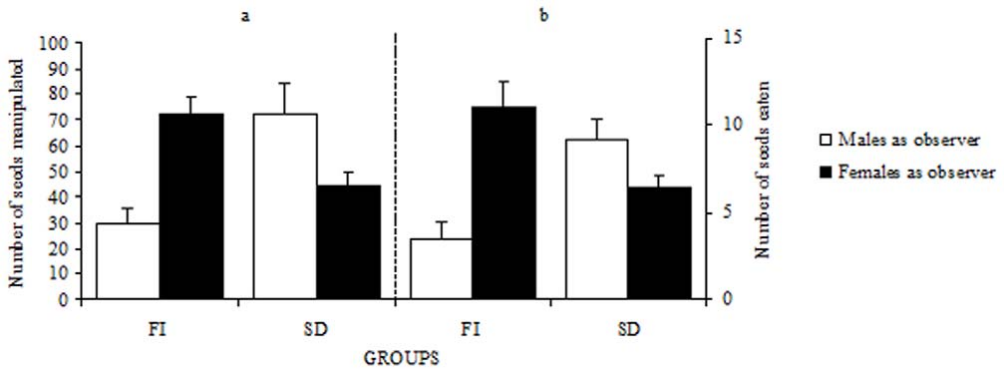
Females demonstrated more in the presence of female demonstrators. Average (±SE) number of seeds picked-up (a) and consumed after husking (b) by a female demonstrator in the presence of an observer either male or female, when pairs of birds interacted freely (FI) and without access to seeds by the observer (SD).

Female demonstrators only accessed sunflower seeds in the FI and SD conditions. In these cases, their behavior was only affected by the sex of the observer as they picked up more seeds when accompanied by a female than by a male (Fig. 4a; *F*_1, 36_ = 4.82, *P*<0.03). A significant interaction between sex and condition (*F*_1, 36_ = 25.2, *P*<0.03) was found. In the FI condition, female demonstrators manipulated less seeds in the presence of a male than of a female observer (Scheffé test: *P*<0.0001) while no differences were found in the SD condition (Scheffé test: *P*>0.05). Similarly, female demonstrators husked and ate more seeds in front of a female than of a male observer (*F*_1, 36_ = 6.78, *P*<0.01). Post-hoc analyses showed that this effect was significant in the FI (Scheffé test: *P*<0.002) but not in the SD condition (*P*>0.05).

**Figure 5:**
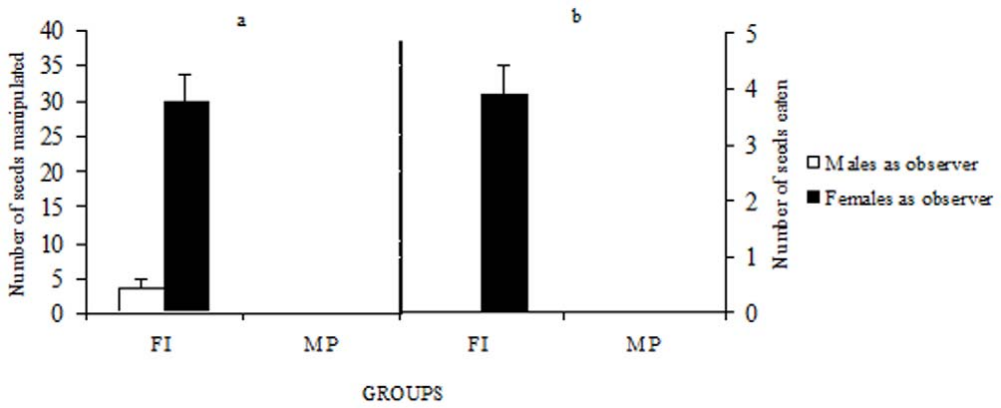
Free interactions with females favored handling only in female observers. Average (±SE) number of seeds picked up (a) and consumed after husking (b) by observers either male or female interacting freely with a female demonstrators (FI) and when a female was merely present (MP).

The behavior of observers confronted with female demonstrators varied depending on their sex (Fig. 5a). The number of seeds manipulated by female observers was significantly higher than that picked up by male observes in the FI condition (*t*_18_ = 7.11, *P*<0.0001). In this condition, all females and six males out of ten manipulated seeds. In the MP condition, naïve birds exposed to a non-demonstrating female did not pick up seeds. In the FI condition, all female observers consumed sunflower seeds under the influence of female demonstrators. No male observer husked any seeds (Fig. 5b).

In general, a high level of tolerance was observed in the FI condition of this experiment compared to the previous one in which males acted as demonstrators. A single attack was observed from a female demonstrator towards a male observer. No aggressive behavior occurred between females. During interactions between female demonstrators and male observers, no motor coordination was observed. In contrast, simultaneous visits to the feeder were observed during interactions between female demonstrators and female observers. The time spent together at the feeder by a female demonstrator and a female observer amounted 53.5±10.42% of the total time spent visiting the feeder. As in the previous section (see ‘Males as demonstrators’) female observers ingested pieces of kernel dropped by female demonstrators (10 out of 10). Two out of ten males were observed ingesting pieces of kernel.

**Figure 6:**
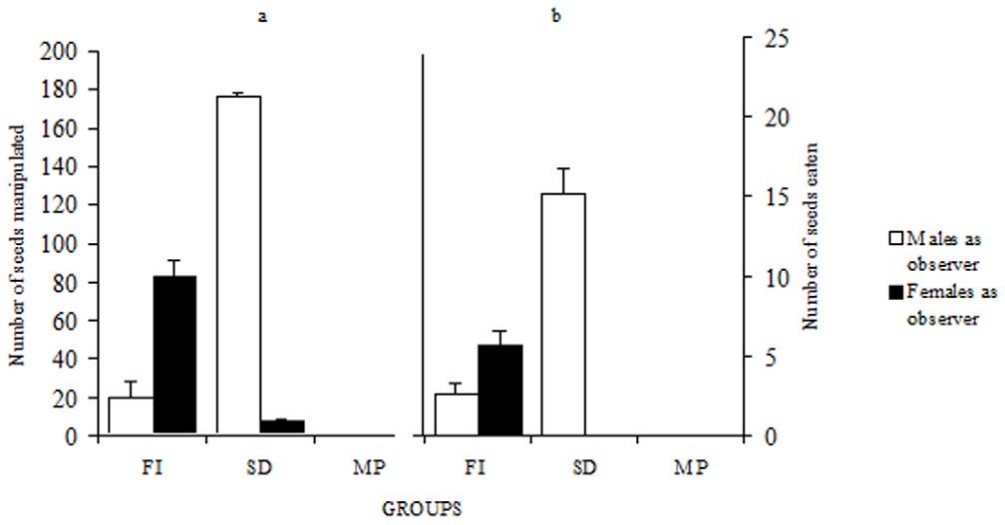
Complete social transmission from females occurred through different pathways in males and in females. Average (±SE) number of seeds picked up (a) and consumed after husking (b) by isolated male or female birds following the various interactions with a female demonstrator, when birds had previously interacted freely with the demonstrator (FI), after visual interactions (SD) and after access to seeds in the mere presence of a bird (MP). Free interactions were necessary for females, while visual interactions were sufficient for males.

When observers were subsequently isolated to check social transmission, only males and females previously exposed to a female demonstrator in action (FI and SD conditions) manipulated seeds. No bird picked up sunflower seeds following the MP condition. All males picked up seeds following FI and SD conditions while ten and eight females did it after the FI and SD condition, respectively. Two-way ANOVA revealed that males picked-up more seeds than females (*F*_1, 36_ = 31.13, *P*<0.0001; Fig. 6a). The interaction between sex and experimental condition was significant (*F*_1, 36_ = 22.18, *P*<0.0001). Indeed, males manipulated more seeds following the SD condition (*P*<0.0001) while females manipulated more seed following the FI condition (*P*<0.001).

Observing a female demonstrator behind a transparent barrier (SD condition) did neither promote husking nor consumption of seeds in female observers. Consumption by females occurred only following the FI condition (9 out of 10 females) while males ingested seeds following both the FI (9 out of 10 males) and the SD condition (10 out of 10 males). One–way ANOVA showed that seed husking varied depending on the experimental condition (F_2, 29_ = 30.84, P<0.0001; Fig. 6b). Indeed, females husked more seeds than males after free interactions with the female demonstrator (Scheffé test: *P*<0.002) while males husked more seeds after visual interactions with the female demonstrator (Scheffé test: P<0.001). These results thus indicate that free access to the target with the female model is required for social transmission of feeding habits to female observers. Male observers, on the other hand, may acquire the novel feeding habit through visual interactions with the female model. The fact that birds of both sexes did neither differ in age, nor in weight or bill size, confirms that differences in social transmission from female demonstrators to male and female observers were due to observer sex and not to other spurious factors.

#### Comparing demonstrator success in terms of seed manipulation and consumption

To compare male and female demonstrators in terms of their demonstrating efficiency, we analyzed seed manipulation and consumption by observers in all conditions taken together.

In terms of manipulation, male demonstrators were more successful than female demonstrators to transmit the feeding habit. Indeed, more observers manipulated seeds following interactions of all three types (FI, SD and MP) with a male than with a female demonstrator (58/60 for male demonstrators; i.e. 97%; 38/60 for female demonstrators; i.e. 63.33%; 2×2 Chi-square after Yates correction = 18. 80, P<0.0001).

In terms of *consumption*, however, the picture changes dramatically. Taking those observers that manipulated seeds as reference, we found that male demonstrators induced consumption in 21 out of 58 manipulators (see above) (36.2%) while female demonstrators did it in 28 out of 38 manipulators (73.68%; 2×2 Chi-square after Yates correction = 11.05, P<0.0007). This confirms that males were not the best demonstrators despite their innovative tendencies and the fact that they induced more seed manipulation.

## Discussion

We studied innovative feeding behavior in canaries, which do not spontaneously consume sunflower seeds. We determined which sex was more innovative and incorporated these seeds to their diet and studied if and how this newly acquired feeding habit was socially transmitted. Sunflower-seed consumption only occurred in males during a familiarization period. In contrast, females rarely ate this seed, thus showing a clear sex dependency of feeding habit innovation. Factors like age, weight or variations in individual strength did not account for this result (Table 1). A decrease in neophobia [29] cannot explain the acquisition of the new feeding habit as males and females manipulated during the familiarization conditions and only males learned how to husk sunflower seeds.

Are innovative males the best demonstrators ensuring new feeding habit transmission towards naïve birds in the population? The answer is not. Social transmission of handling skills between individuals depended both on experimental conditions and on the sex of the observers. In male-male free interactions, experienced males proved to be bad demonstrators due to their aggressive behavior towards male observer. Only when a transparent partition prevented such aggression did male observers learn from an experienced male after visual interactions. In contrast, female observers learned to husk sunflower seeds during free interactions with an experienced male, while they did not learn through visual interactions. During free interactions, aggressive behavior between males limited social transmission. Indeed, the observer gets fewer demonstrations of seed manipulation or husking from a male demonstrator engaged in aggressive behavior. In addition, aggressive interactions between males distract the observer. Similar results have been found in zebra finches [13]. Observers could counterbalance the negative influence of aggression by limiting interactions and just observing the demonstrator's behavior. However, experienced males tend to push naïve birds of the same sex away from the food source so that observing becomes difficult.

Males seem to be more innovative because they are bolder than females. In birds boldness is a personality trait linked with aggressive behavior [30]. In male and female observers, demonstration of eating seeds was required to learn this new skill (situation FI and /or SD). No bird learned handling following the MP condition. The mere presence of a non-demonstrating male only familiarizes birds of each sex with sunflower seeds that are picked up but not consumed (reduction of neophobia by presence of a conspecific [31]). Ability of males to learn from a demonstrator (male or female) placed behind a transparent barrier (SD) or from a female demonstrator in the FI condition resulted from familiarization with sunflower seeds by stimulus enhancement (recognition of an object manipulated independently of its location [32]–[34]). Attention of male observers was drawn to the novel seed giving it a positive value. This familiarization rapidly leads the male observers to handle sunflower seeds after their isolation because of their boldness. The manipulation of a great amount of seeds by males suggests individual learning of handling by trials and errors.

In contrast to males, female canaries that acquired the new feeding habit were good demonstrators due to their higher tolerance towards observers either males and females. Female observers, less aggressive than males, were not bold enough to handle successfully large seeds such as those from sunflower. In the visual interaction condition (SD), social transmission was limited to an increase of seed manipulation compared to the MP condition (absence of demonstration). Complete social transmission only occurred after free interactions (FI) with a demonstrator either male or female. In these cases, females could access seeds with the demonstrator and could eat pieces of kernel dropped from the demonstrator's bill. In addition, motor coordination was possible between partners (effect of social facilitation [32]). Access to reward by local enhancement and social facilitation [35] may thus explain the complete social transmission in the FI condition as female observers successfully husked sunflower seeds after isolation. A similar result was found by Aisner and Terkel [36] who studied social learning of pine cone striping in rats (transmission from mother to juveniles). In this case, simultaneous access to reward with the mother was necessary for the young to perform the task, thus underlining the importance of encountering seeds that have been partially husked and eaten by the demonstrator. Similar facilitating effects have been found in other mammals and birds [20]–[23], [37]. Our results show that social relationship is a crucial factor for the spread of a new feeding habit in the population. Coussi-Korbel and Fragazy [12] underlined that the demonstrator's tolerance towards the observer, simultaneous access to the food source and motor coordination between partners are all required for facilitating feeding habit transmission. In our experiments, these conditions were met in the FI condition involving a male demonstrator and a female observer or a female demonstrator and a female observer. In these cases, birds accessed the food simultaneously due to higher tolerance and motor coordination was enhanced by the high activity of the demonstrators. In the other two remaining conditions, male demonstrator and male observer and female demonstrator and male observer, we did not observe facilitated feeding habit transmission. In the first case, as mentioned above, aggressive behavior from the male demonstrator towards the male observer prevented such transmission. In the second case, females exhibited a low level of demonstration despite the absence of aggressive interactions. It seems that the presence of the male disturbed the feeding activity of the female demonstrator. This thus impaired learning by observer males.

We conclude that females play an important role in spreading the novel feeding behavior while bold males are more innovative but less effective as demonstrators at least to other males. This result is confirmed by the comparison between overall numbers of bird which learned from male and female demonstrators. Whereas, more birds of both sexes tended to pick up seeds after interacting with males, females were more effective demonstrators for handling skills than males. Thus, the most innovative individuals are not the best demonstrators. Transmission of skills often demands periods of free interaction between subjects and these periods involve sex-dependent aggressive behaviors, thus confirming the non-randomness of the spread of feeding habits within a population [13], [38]–[40].

Taking into account the organization of social behavior and social structure of canaries allows appreciating the natural framework in which the effects studied in our work operate. Wild canaries live in islands in which food sources are scarce and distributed in patches. They do not possess a preference for specific plants in contrast to specialists. Birds search their food in flocks having more males than females [41]. This situation promotes, therefore, competition for food sources, but may also favour social learning due to interactions within flocks. Moreover, wild canaries are monogamous and form pairs that are maintained even in non-breeding seasons and throughout several years [42]. In this context, being able to innovate feeding habits and to access faster novel foods may confer additional advantages: males can spend more time singing to attract females and breed. Given that bi-parental care is essential for offspring survival, it is possible to understand the role of both partners with respect of their juveniles. While males will be more innovative and transmit their feeding skills to their female partner, females will spread these novel skills within the population. Thus, males and females play a complementary role in the exploitation of novel food sources.
